# Acute Concurrent Cardiocerebral Infarction Associated With Trousseau Syndrome: A Case Report

**DOI:** 10.7759/cureus.77642

**Published:** 2025-01-18

**Authors:** Aika Okubo, Shunya Sugai, Kyoko Morikawa, Keiichi Tsuchida, Takumi Kurabayashi

**Affiliations:** 1 Cardiology, Niigata City General Hospital, Niigata, JPN; 2 Obstetrics and Gynecology, Niigata University Medical and Dental Hospital, Niigata, JPN; 3 Obstetrics and Gynecology, Niigata City General Hospital, Niigata, JPN

**Keywords:** cardiocerebral infarction, ischemic stroke, myocardial infarction, trousseau syndrome, uterine cancer

## Abstract

This case report describes a 66-year-old woman presenting with acute aphasia and right hemiplegia caused by multiple cerebral infarctions. Diagnostic evaluation confirmed an asymptomatic myocardial infarction (MI), as evidenced by elevated troponin I levels (3.1 ng/mL); ST-segment elevation in leads II, III, and aVF on electrocardiography; and coronary angiography showing a thrombotic occlusion in the distal portion of the left anterior descending artery. Elevated tumor markers and imaging identified stage IB endometrioid uterine carcinoma. She had a malignant tumor and multiple thrombotic events and was diagnosed with Trousseau syndrome. Management involved hysterectomy with bilateral salpingo-oophorectomy followed by chemotherapy and anticoagulation therapy, initiated with heparin and transitioned to apixaban due to the desire to minimize impact on the quality of life by using an oral medication. The patient achieved a favorable recovery without recurrent thrombotic events. To the best of our knowledge, this is the first report of a patient with Trousseau syndrome associated with stage IB uterine cancer developing acute cardiocerebral infarction (CCI). This case highlights the importance of identifying Trousseau syndrome in patients with multiple cerebral infarctions and underscores the need to screen for and asymptomatic MI, even in the absence of cardiac symptoms.

## Introduction

Trousseau syndrome is defined as one or more thrombotic events of unknown cause that precede or accompany diagnosis of malignancy [1]. It has been suggested that Trousseau syndrome is more likely to occur in tumors that produce mucin, which is one of the factors that can promote blood coagulation and lead to ischemic stroke. Fourteen percent of patients with cancer have evidence of cerebrovascular disease events [2]. The term cardiocerebral infarction (CCI) was proposed by Omar et al. [3] and denotes the simultaneous or metachronous occurrence of ischemic stroke and myocardial infarction (MI). It is very rare, with reported incidence being 0.009% of ischemic strokes [4]. The low incidence rates may be due to underreporting, given the diagnostic difficulty of this condition [5]. Patients with CCI may have difficulty recognizing symptoms of MI due to cognitive impairment and complications such as aphasia.

Here, we report a rare case of CCI associated with Trousseau syndrome caused by uterine cancer.

## Case presentation

A 66-year-old woman presented with aphasia and right hemiplegia. She had noted no health-related changes prior to the previous day, was healthy, and had no notable medical history, including blood coagulopathy. In addition, she was nulliparous.

Physical examination revealed a Glasgow Coma Scale score of 12 (E4V2M6), temperature of 36.8°C, blood pressure of 232/107 mmHg, and pulse rate of 97/min. The National Institutes of Health Stroke Scale score was 13. She spoke only a few words and had dysarthria. She could not maintain elevation of the right upper extremity.

**Figure 1:**
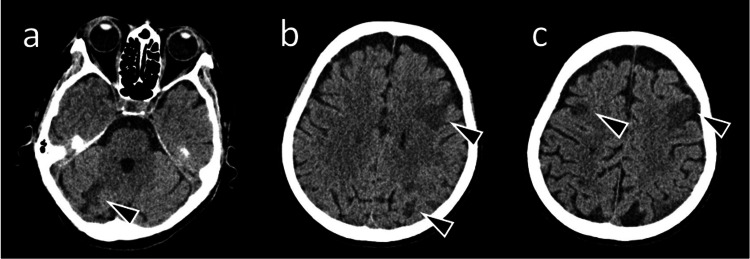
Brain computed tomography image Computed tomography image without contrast showing multiple low absorption areas (arrowheads). Hypodense areas are observed in (a) the right cerebellar hemisphere, (b) the left fronto-parietal lobe and left occipital lobe, and (c) the left fronto-parietal lobe and right frontal lobe. These indicate areas of infarction.

Blood tests showed severe anemia and elevated D-dimer levels (Table 1), suggesting a hypercoagulable state. Acute cerebrovascular disease was suspected and a brain computed tomography (CT) scan performed immediately. This revealed multiple cerebral infarctions, predominantly affecting the left lobe (Figure 1), which were considered the cause of aphasia and right hemiplegia. 

**Table 1:** Blood test results ALP: alkaline phosphatase, ALT: alanine aminotransferase, AST: aspartate aminotransferase, BUN: blood urea nitrogen, CK: creatine kinase, Cl: chlorine, Cre: creatinine, CRP: C-reactive protein, Hb: hemoglobin, K: potassium, LDH: lactate dehydrogenase, Na: sodium, Plt: platelets, RBC: red blood cells, T-bil: total bilirubin, TP: total protein, WBC: white blood cells

Test	Results/units	Normal range
WBC	6000 /μL	3300-8600
RBC	4.06 ×10^6^/μL	3.86-4.92
Hb	6.3 g/dL	11.6-14.8
Plt	200 ×10^4^/μL	158-348
D-dimer	5.8μg/mL	<1.0
AST	47 U/L	13-30
ALT	16 U/L	7-23
ALP	225 U/L	106-322
LDH	348 U/L	124-222
CK	265 U/L	41-153
TP	9.0 g/dL	6.6-8.1
T-Bil	1.0 mg/dL	0.4-1.5
BUN	15.9 mg/dL	8-20
Cre	0.57 mg/dL	0.46-0.79
Na	138 mmol/L	138-145
K	4.0 mmol/L	3.6-4.8
Cl	101 mmol/L	101-108
CRP	1.15 mg/dL	<0.14

**Figure 2:**
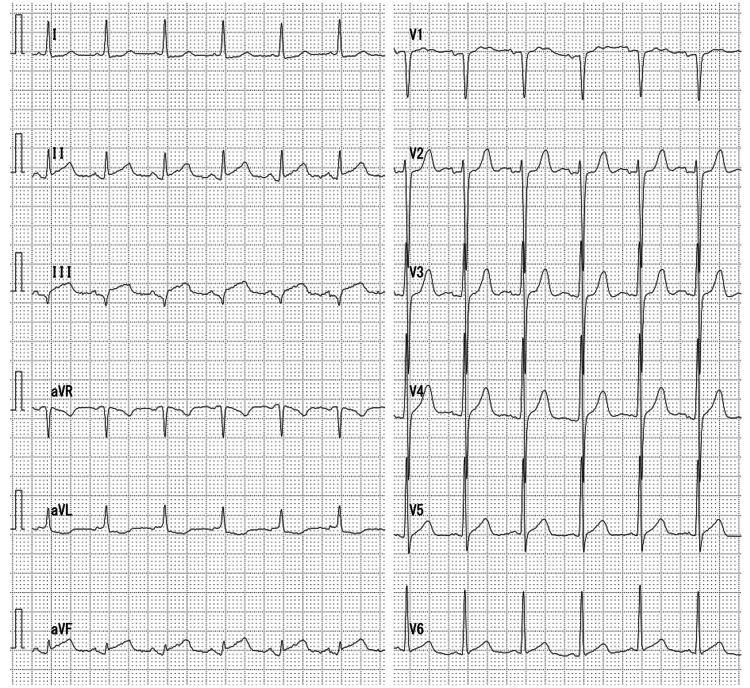
Electrocardiogram findings Electrocardiogram showing normal sinus rhythm, narrow QRS, no axis deviation, and ST-segment elevation in leads II, III, and aVF with reciprocal change in leads I and aVL.

Screening electrocardiography on admission revealed normal sinus rhythm and ST-segment elevation in leads II, III, and aVF with reciprocal changes in leads I and aVL (Figure 2). Transthoracic echocardiography showed hypokinesis of the left ventricular apex with no evidence of intracardiac thrombus. Additional cardiac biomarker testing showed troponin I of 3.1 ng/mL (normal: <0.016 ng/mL). Although she reported no chest pain, the concomitant ST elevation and elevated troponin levels were highly suggestive of MI. Based on the electrocardiogram and blood test results, the MI was considered to be in the acute phase, and the CCI was assumed to have occurred almost simultaneously.

**Figure 3:**
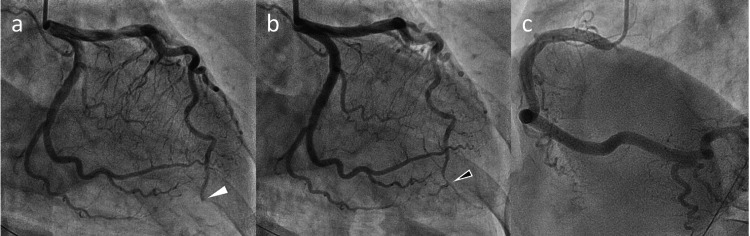
Coronary angiography before and after the procedure (a) Pre-procedural coronary angiogram showing total occlusion of the distal left anterior descending artery (white arrowhead). (b) Post-procedural coronary angiogram showing successful recanalization of the occluded vessel following thrombus aspiration (black arrowhead). The main component of the extracted thrombus was fibrin. (c) Right coronary angiogram demonstrating no stenotic lesions.

Because she had presented more than 24 hours after the onset of symptoms, we considered that intravenous thrombolysis and endovascular therapy were not indicated for her cerebral infarction. Emergency coronary angiography revealed a thrombotic occlusion in the distal left anterior descending artery (Figure 3a), leading to the diagnosis of acute apical MI. A primary percutaneous coronary intervention using an aspiration device to eliminate the thrombotic debris was successful in achieving recanalization of the occluded artery (Figure 3b). The patient was hemodynamically stable after achieving coronary reperfusion with a mild increase in creatine kinase myocardial band (peak concentration = 69 U/L). Histological examination of the aspirated material showed red thrombi with no atheromatous components.

In the acute phase, blood pressure management and rehabilitation were initiated, along with the administration of unfractionated heparin, while edaravone injections were used for cerebral protection. Transesophageal echocardiography revealed no large thrombus in the left ventricle but identified a patent foramen ovale, while CT confirmed deep vein thrombosis in the lower extremities.

**Figure 4:**
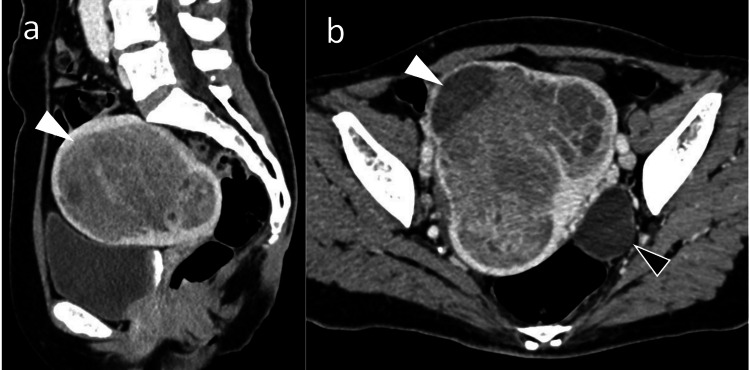
Pelvic computed tomography image Contrast-enhanced computed tomography images showing a heterogeneously enhanced mass (white arrowhead) within an enlarged uterus. There is also a left ovarian cyst that is not suspicious of malignancy (black arrowhead). (a) Sagittal sectional image; (b) axial sectional image.

We considered the multiple cerebral infarctions suspicious of Trousseau syndrome and therefore performed extensive screening for occult cancer. Gynecological examination disclosed genital bleeding. A whole-body CT scan and magnetic resonance imaging (Figure 4) showed uterine enlargement and a left ovarian cyst with no enlargement of lymph nodes or evidence of distant metastasis. We accordingly suspected uterine cancer. Tumor marker testing revealed very high concentrations of carbohydrate antigen 125 (CA125; 1,548 U/mL, normal: 35 U/mL) and carbohydrate antigen 19-9 (CA19-9; 57,023 U/mL, normal: 37 U/mL). Endometrial biopsy demonstrated atypical endometrial hyperplasia; however, the CT imaging findings were highly suggestive of malignancy. We therefore performed bilateral salpingo-oophorectomy and hysterectomy. Histopathological assessment of the operative specimen revealed endometrioid carcinoma (Grade 1), which was classified as Stage IB. The risk of postoperative recurrence was intermediate, and after consultation with the patient, we decided to administer postoperative adjuvant chemotherapy consisting of docetaxel and carboplatin. A direct oral anticoagulant (apixaban) was substituted for her intravenous heparin postoperatively.

She was discharged after a 75-day hospitalization. Four months after surgery, the tumor marker concentrations had decreased significantly (CA125, 4.7U/mL; CA19-9, 42.3U/mL), with no recurrence observed. She continues to take oral apixaban for secondary prevention of the CCI. Despite persistent right hemiplegia and ongoing rehabilitation, her condition has remained good with no recurrence of uterine cancer or further ischemic events. 

## Discussion

To the best of our knowledge, this is the first report of a patient with Trousseau syndrome associated with stage IB uterine cancer developing acute CCI. CCI is quite rare but can be life-threatening and therefore requires prompt medical intervention. No evidence-based treatment plan for CCI has been established, and given the potential for various comorbidities, it is crucial for a multidisciplinary team (neurology, cardiology, and oncology) to collaborate and make timely decisions regarding the treatment strategy. Fortunately, the present case had a favorable outcome, which was made possible through effective collaboration among the multidisciplinary team.

Trousseau syndrome is defined as thrombotic events in patients with an underlying malignancy. Although there are multiple possible mechanisms for the development of this syndrome [1], it is commonly associated with mucin-producing tumors. Mucin is known to stimulate blood coagulation through adhesion-dependent reciprocal activation of neutrophils and platelets [6,7]. Concentrations of the markers CA125 and CA19-9 may be high in individuals with mucin-producing tumors. In our patient, they were very high despite her having a stage IB uterine cancer. Her risk of thromboembolism was therefore considered to be high.

The possible causes of our patient’s CCI are as follows. Her multiple localized thrombi may have been attributable to hypercoagulability. Alternatively, she may have had an undetected source of thrombus in the left ventricle, such as nonbacterial thrombotic endocarditis. It is also possible that she had a deep vein thrombosis that could have resulted in paradoxical embolism through her patent foramen ovale.

Heparin is recommended for treating Trousseau syndrome [8]. We switched our patient from heparin to apixaban because the latter has been shown to be effective in venous thromboembolism associated with cancer [9] and because oral agents have minimal impact on quality of life. However, little data are available on the efficacy of apixaban in Trousseau syndrome. Further studies are needed to investigate this. Such patients should be followed up carefully.

In this case, although the risk of thrombotic exacerbation associated with chemotherapy was a concern, thrombotic events were effectively managed with anticoagulant therapy. Consequently, the decision was made to proceed with postoperative chemotherapy. Paclitaxel and carboplatin are commonly used in postoperative adjuvant chemotherapy for uterine cancer. However, paclitaxel can cause peripheral neuropathy, so it is difficult to use them after cerebral infarction as in this case. Docetaxel with platinum is reported to have same expected efficacy [10] and we used them. Long-term follow-up, with attention to the recurrence of thrombosis and malignancy, will be necessary in the future.

One of the characteristics of this case was the absence of chest pain in spite of a documented MI. Sakuta et al. reported a 55-year-old woman with stage IV ovarian cancer who developed CCI and had an elevated CA-125 level (190 U/mL) [11]. She was noted to have a painless MI, similar to the present case. Some individuals with MI have atypical symptoms without chest pain. About half of reported patients with unrecognized MI had no symptoms, the other half having non-specific symptoms [12]. The risk factors for MI with atypical symptoms include older age, being female, cardiovascular disease (including stroke), chronic kidney disease, and diabetes mellitus [13]. Patients with cerebral infarction and Trousseau syndrome are at high risk of unrecognized MI, as in the present case. Even when chest pain is present, patients with aphasia may be unable to report it. Therefore, this case underscores the importance of recognizing Trousseau syndrome and highlights the need for screening for asymptomatic MI with electrocardiography and cardiac biomarkers, even in the absence of typical cardiac symptoms.

## Conclusions

We report a rare case of CCI caused by Trousseau syndrome associated with uterine cancer. The absence of chest pain in this case highlights the difficulty of recognizing MI in patients with aphasia or other cognitive impairments, a challenge that clinicians must be prepared to address. When evaluating a patient with stroke due to Trousseau syndrome, it is essential to be alert for concurrent thromboembolic events, including MI, which can occur without typical symptoms, particularly in patients with neurological deficits. There is a paucity of published data on standardized treatment protocols for CCIs, and the accumulation of future cases is necessary to better manage CCIs.

## References

[1] Varki A (2007). Trousseau's syndrome: multiple definitions and multiple mechanisms. Blood.

[2] Bao L, Zhang S, Gong X, Cui G (2020). Trousseau syndrome related cerebral infarction: clinical manifestations, laboratory findings and radiological features. J Stroke Cerebrovasc Dis.

[3] Omar HR, Fathy A, Rashad R, Helal E (2010). Concomitant acute right ventricular infarction and ischemic cerebrovascular stroke; possible explanations. Int Arch Med.

[4] Yeo LL, Andersson T, Yee KW (2017). Synchronous cardiocerebral infarction in the era of endovascular therapy: which to treat first?. J Thromb Thrombolysis.

[5] Ng TP, Wong C, Leong EL (2022). Simultaneous cardio-cerebral infarction: a meta-analysis. QJM.

[6] Wahrenbrock M, Borsig L, Le D, Varki N, Varki A (2003). Selectin-mucin interactions as a probable molecular explanation for the association of Trousseau syndrome with mucinous adenocarcinomas. J Clin Invest.

[7] Shao B, Wahrenbrock MG, Yao L (2011). Carcinoma mucins trigger reciprocal activation of platelets and neutrophils in a murine model of Trousseau syndrome. Blood.

[8] Lee AY, Levine MN, Baker RI (2003). Low-molecular-weight heparin versus a coumarin for the prevention of recurrent venous thromboembolism in patients with cancer. N Engl J Med.

[9] Agnelli G, Becattini C, Meyer G (2020). Apixaban for the treatment of venous thromboembolism associated with cancer. N Engl J Med.

[10] Nomura H, Aoki D, Michimae H (2019). Effect of taxane plus platinum regimens vs doxorubicin plus cisplatin as adjuvant chemotherapy for endometrial cancer at a high risk of progression: a randomized clinical trial. JAMA Oncol.

[11] Sakuta K, Mukai T, Fujii A, Makita K, Yaguchi H (2019). Endovascular therapy for concurrent cardio-cerebral infarction in a patient with Trousseau syndrome. Front Neurol.

[12] Kannel WB, Cupples LA, Gagnon DR (1990). Incidence, precursors and prognosis of unrecognized myocardial infarction. Adv Cardiol.

[13] Fujino M, Ishihara M, Ogawa H (2017). Impact of symptom presentation on in-hospital outcomes in patients with acute myocardial infarction. J Cardiol.

